# Does laser treatment affect outcome in pilonidal sinus disease? Long-term multicenter retrospective analysis of pit-picking alone vs. pit-picking with laser

**DOI:** 10.1007/s10151-026-03314-8

**Published:** 2026-05-13

**Authors:** N Cigdem Arslan, Ismail Ahmet Bilgin, Baris Gulcu, Baris Bayraktar, Ismail Cem Eray, Tayfun Bisgin, Yasemin Yildirim, Afag Aghayeva, Ishak Aydin, Cagri Bilgic, Salih Boluk, Cahide Inci Kurtul, Nur Ramoglu, Ali Yildirim, Ilknur Erenler Bayraktar, Onur Bayraktar

**Affiliations:** 1 Department of Surgery, Istanbul Medipol University, Istanbul, Turkey; 2 Department of Surgery, Acibadem Mehmet Ali Aydinlar University, Istanbul, Turkey; 3 Department of Surgery, Medicana Bursa Hospital, Bursa, Turkey; 4 Department of Surgery, Medical Park Gebze Hospital, Gebze, Turkey; 5 Department of Surgery, Cukurova University, Adana, Turkey; 6 Department of Surgery, Dokuz Eylul University, İzmir, Turkey; 7 Department of Surgery, Istanbul Bilim University, Istanbul, Turkey; 8 Department of Surgery, Liv Hospital Vadi Istanbul, Istanbul, Turkey; 9 Department of Surgery, Istanbul Biruni University, Istanbul, Turkey; 10 Department of Surgery, Istanbul Health and Technology University, Hakki Yeten Cad. No:13/68, 34394 Sisli, Istanbul, Turkey

**Keywords:** Pilonidal sinus disease, Pit-picking, Laser treatment, Recurrence

## Abstract

**Objectives:**

The optimal management of pilonidal sinus disease (PSD) remains controversial, with multiple treatment approaches available. Pit-picking is a minimally invasive technique, often enhanced with laser treatment (LT), but the long-term benefits of LT remain uncertain. The objective of this study is to compare pit-picking alone versus pit-picking combined with LT.

**Methods:**

This is a multicenter retrospective cohort study including seven centers across Turkey. Patients who underwent pit-picking surgery for PSD between June 2017 and March 2025 were included. Patients receiving adjunctive treatments beyond LT, undergoing excisional procedures, or with incomplete follow-up data were excluded. Pit-picking surgeries performed with or without LT were compared. The primary measure was the recurrence rate at 5 years. Secondary outcomes were postoperative pain, time to complete healing, complications, return to work, and costs.

**Results:**

Of 306 patients, 109 (35.6%) underwent pit-picking alone, and 197 (64.4%) received pit-picking with LT. The complication rate was lower in the LT group (6.1% vs. 14.7%, *p* = 0.012). Patients treated with LT had shorter times to pain-free sitting (median 5 vs. 7 days, *p* < 0.001) and return to work (3 vs. 6 days, *p* < 0.001). Complete healing was achieved in 97.4% of patients, with a median time of 14 days. Recurrence rates at 5 years were similar (pit-picking: 13.8%, pit-picking + LT: 12.7%, *p* = 0.460). Costs were higher for the LT group ($1212 ± 146 vs. $888 ± 148), although complicated pit-picking cases had comparable costs to the LT group ($1198 ± 370, *p* = 0.004). Risk factors for recurrence included high BMI, family history, advanced Tezel stage, and postoperative complications.

**Conclusions:**

While LT improves early postoperative outcomes, it does not impact long-term recurrence rates. The increased cost of LT should be weighed against its benefits in reducing complications, pain, and recovery time. Further randomized trials are needed to refine patient selection criteria and assess cost-effectiveness.

**Clinical trial registration:**

NCT05569135, Registration date: 05.10.2022.

## Introduction

The optimal management of pilonidal sinus disease (PSD) remains controversial, with numerous surgical and conservative treatment modalities currently available. Although surgical excision is widely accepted as the standard approach for chronic PSD [1, 2], there is a growing demand among both surgeons and patients for outpatient-based treatments.

Since Bascom [3] introduced the cleft lift technique, minimally invasive approaches have evolved, incorporating slight modifications of pit-picking [4]. The core principle of pit-picking and its variations are excision or curettage of diseased tissue and debris through very limited incisions, preserving as much healthy skin as possible. Advancements, including laser and endoscopic technologies, along with adjunctive applications like phenol and fibrin glue, have been integrated into this procedure to further enhance clinical outcomes [5–7].

Most outcomes related to pit-picking are based on procedures that combine it with other approaches. A few studies on pit-picking alone reported recurrence rates of 10–51% at 12–83-month follow-up [8, 9]. Recently, laser treatment (LT) has been proposed as an adjunct to pit-picking, offering reduced bleeding, low postoperative pain, and improved wound healing. In a review, primary healing after LT has been reported to be 94.4%, and the pooled recurrence rate at a median of 12 (7–25) months was 3.8% [5]. However, existing studies are limited by short follow-up durations, and it remains uncertain whether the observed clinical effectiveness comes primarily from the addition of LT or from the pit-picking itself [10].

To address these issues, we carried out a retrospective single-center audit in 2023 and published the 36-month recurrence rates for pit-picking and LT [11]. We observed that combining LT with pit-picking positively influenced early postoperative outcomes; however, no significant impact on recurrence rates was identified. Consequently, the current multicenter study was designed to include a larger cohort.

The primary aim of this study is to compare pit-picking and pit-picking combined with LT regarding early postoperative outcome, long-term recurrence, and costs.

## Methods

This multicenter retrospective study analyzed patients who underwent pit-picking surgery at seven high-volume centers across Turkey. The study received ethical approval from the Istanbul Medipol University Ethics Committee (approval no. E-10840098–772.02–2926). The pilot audit study [11] was registered on ClinicalTrials.gov (ID: NCT05569135). Due to its retrospective design, individual informed consent was not required; however, all patients had previously provided surgical consent, which included permission for the anonymized use of their recorded data in scientific research. This study was conducted in accordance with the Strengthening the Reporting of Observational Studies in Epidemiology (STROBE) guidelines.

### Patients

**Fig. 1:**
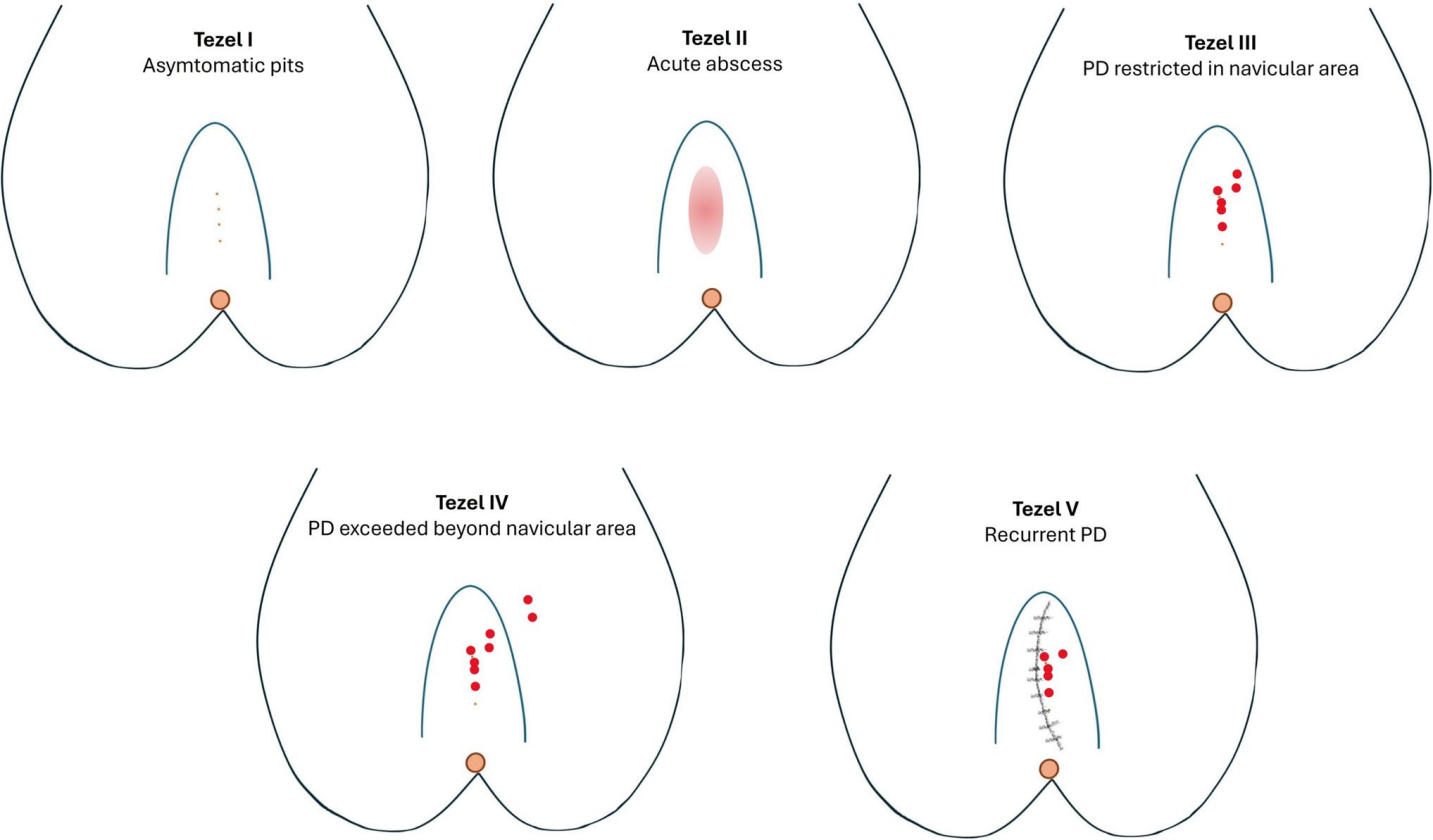
Illustration of the Tezel navicular area classification

Data of the patients who underwent surgery for PSD between June 2017 and March 2025 were reviewed. The inclusion criteria were patients > 18 years old having a pit-picking operation with or without the addition of LT. Exclusion criteria included any adjunct methods to pit-picking other than LT (e.g., EPSIT, phenol, fibrin glue) as well as excisional procedures and incision and lay-open techniques. The disease was classified according to the Tezel classification (Fig. 1) [12]. Patients with asymptomatic pits (Tezel I) and acute abscesses (Tezel II) were excluded. Hospital electronic records, nurse observation sheets, and outpatient clinic archives were reviewed. Patients without complete data on the primary outcome (5-year recurrence) and key secondary outcomes (early complications, return to work, and time to complete healing) were excluded from the analysis. However, patients with missing data on day-to-painless-sitting and visual analog scale (VAS) scores were not excluded; these analyses were performed within the subgroup of patients with available data. Tezel II patients were included in the analysis if they underwent definitive pit-picking at least 2 weeks after abscess drainage and at least 1 week after cessation of antibiotics.

The patients' peri- and postoperative follow-up data were recorded, and they were enrolled in pit-picking alone or pit-picking with LT. The primary outcome was comparing recurrence rates at 5 years between the two groups. Secondary measures were postoperative complications, time to return to work, time to complete healing, and costs.

### Surgical technique

The LT was first introduced in the participating centers between 2013 and 2018. The principal investigator (CA) jointly participated in PSD procedures with all participating centers during several professional meetings and workshops, and there is consensus among these centers regarding the standardization of the surgical technique. The decision to use LT was based on the surgeon’s or patient’s preference and, in some cases, influenced by reimbursement policies, without specific selection criteria.

The procedures were performed under general, regional, or local anesthesia (with or without sedation) by methods described before [11, 13, 14]. Patients were positioned prone. Sinus openings were identified, and depending on the extent and number of tracts, one to three sinuses were enlarged using a scalpel, a clamp, or a punch biopsy needle. Hair and necrotic tissue were removed using a clamp, curette, or brush. The cavity was irrigated with saline. For patients undergoing LT treatment, a radial laser probe (NeoV® laser, G.N.S neolaser Ltd., Regus, Israel, or Leonardo Dual 45®, Biolitec Biomedical Technology GmbH, Jena, Germany) with a 980 or 1470-nm wavelength was introduced through the pits and used in continuous or pulse mode at 10, 13 or 15 W, with a total of 80–110 J administered per 1 cm of the tract while the probe was gradually retracted. All lateral extensions and sinus tracts were treated. In both groups, a gauze dressing was applied postoperatively. No additional wound care was recommended except for daily showering. Most patients were discharged on the same day, but some who underwent general anesthesia stayed overnight.

### Postoperative follow-up

Patients were discharged with no restriction on sitting and showering immediately after surgery. Follow-up assessments were scheduled at postoperative days 3 and 10, and at 1, 6, and 12 months. Recurrence was evaluated via telephone interviews at 24, 36, and 60 months. Patients were provided with a VAS form to assess pain levels on postoperative days 1, 7, 10, and 30. The same form was used to record the time to painless sitting.

Patients who reported recurrent symptoms during phone interviews were invited for an in-person evaluation to confirm recurrence. Since treatment was not recommended for asymptomatic cases, recurrence was defined based on patient-reported symptoms. Seroma was classified as fluid accumulation within subcutaneous tissue without signs of infection, whereas hematoma was defined as blood or clot accumulation within the subcutaneous layer. Surgical site infection was diagnosed based on purulent drainage or wound dehiscence, with at least one of the following symptoms: pain, tenderness, erythema, swelling, or localized warmth, and, when present, positive culture results [15]. The time to resume daily activities was recorded as the number of days until return to work, and the time to achieve painless sitting was documented separately. Complete healing was defined as full epithelialization of the pits without spontaneous or provoked discharge. Cases where symptoms persisted beyond 2 months postoperatively were categorized as non-healing. Recurrence was defined as the reappearance of symptoms after documented complete healing. However, during recurrence analyses, non-healing patients were included in the recurrence group, as this was a small group and all these individuals underwent subsequent surgical interventions. Time to recurrence was recorded as 2 months for these patients.

### Cost analysis

Due to variations in insurance reimbursement rates among the seven centers, including all centers in our cost analysis, this would not have accurately determined the actual situation. Therefore, the cost analysis was made for patients reflecting the most common practice nationwide—patients receive treatment at private hospitals, covered by public social security, and pay an additional co-payment. Consequently, 133 patients from Istanbul Medipol University were included. All these patients underwent office-based procedures under local anesthesia without sedation.

Total hospital costs included procedure-related charges (office use, local anesthetics, disposable equipment, laser) and 60-day follow-up related charges (office visits, wound care, seroma aspirations, cauterization, etc.). Costs were expressed in US dollars, and due to exchange rate fluctuations, each patient's expenses were calculated and recorded using the Turkish lira-to-US dollar exchange rate on the date the expense was incurred.

### Statistical analysis

Data analysis was conducted using IBM SPSS for Windows v.26. Categorical variables were presented as frequency (*n*) and percentage (%) and compared using the chi-square test or Fisher’s exact test, as appropriate. Continuous variables were assessed for normality using the Shapiro-Wilk test. Normally distributed variables were expressed as mean ± standard deviation and compared using the independent samples *t*-test. Non-normally distributed variables were reported as median (range) and analyzed using the Mann-Whitney U test. A *p*-value < 0.05 was considered statistically significant. Time-to-recurrence was analyzed using Kaplan-Meier survival estimates, with recurrence coded as the event (status = 1) at the date of clinically confirmed recurrence and non-recurrence right-censored (status = 0) at the last documented follow-up (clinic visit or scheduled telephone assessment). Group differences and recurrence-associated factors were compared using log-rank (Mantel-Cox) tests, and Kaplan-Meier curves were presented by treatment group and relevant risk strata (e.g., BMI, Tezel stage, postoperative complications). Follow-up time was measured in months from the index procedure; patients categorized as non-healing at ≥ 2 months were included as events at 2 months, as prespecified. Post hoc power for the primary endpoint was computed using a two-sided z-test for two independent proportions at α = 0.05 based on the observed incidences and sample sizes, with effect size expressed as Cohen’s h (h = 2·arcsin√p1 − 2·arcsin√p2) and variance taken from group-specific proportions under the alternative.

### Use of artificial intelligence

We used Microsoft Co-pilot for grammar and spell-checking.

## Results

**Fig. 2:**
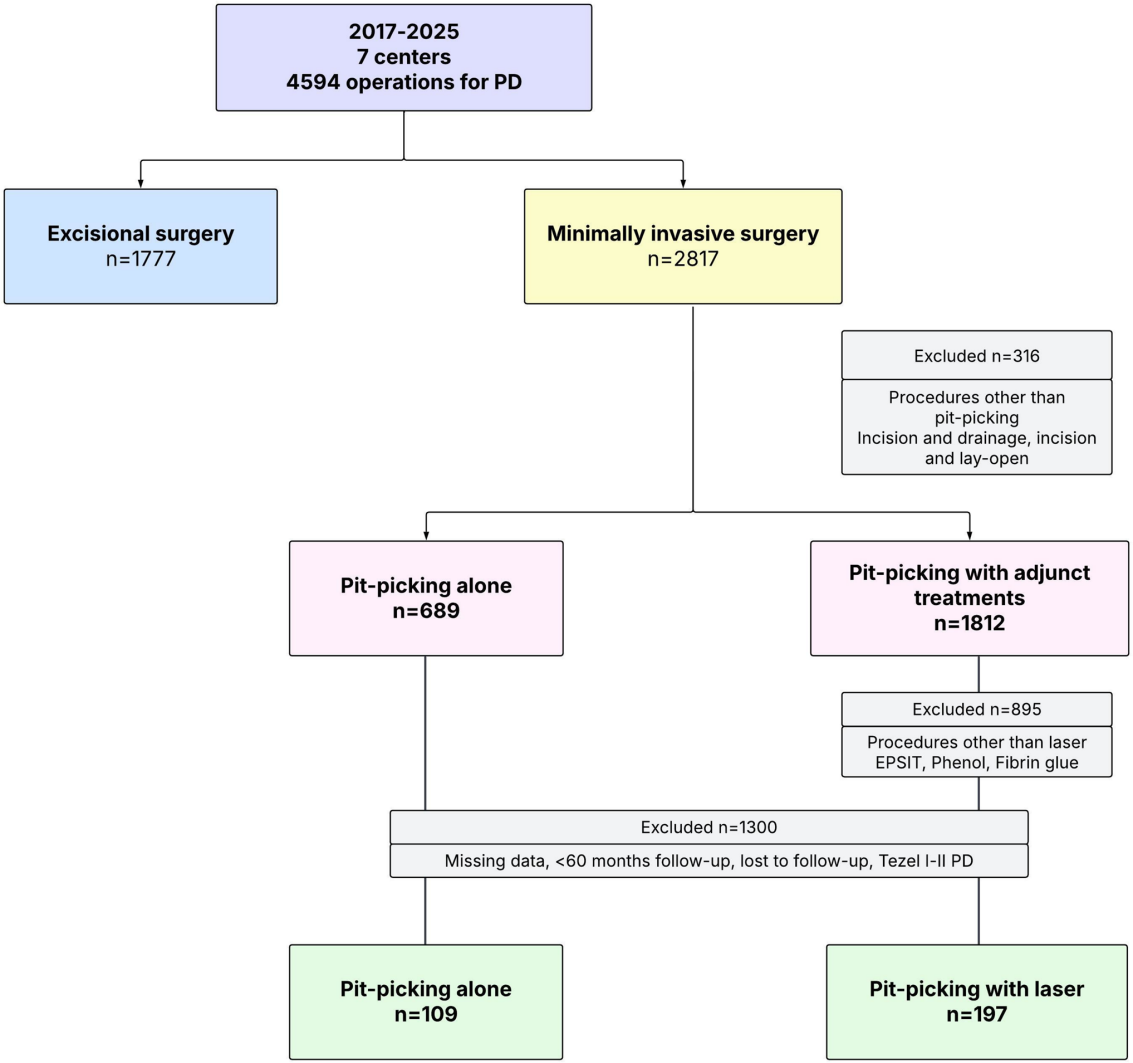
Flow diagram of the study

Between 2017 and 2025, 4594 records were reviewed (Fig. 2). A total of 306 patients were included in the analysis, of whom 109 (35.6%) underwent pit-picking alone, while 197 (64.4%) received pit-picking combined with LT. The distribution of patients across institutions is given in Table 1.

**Table 1:** Distribution of surgeries reviewed between 2017 and 2025 across centers and patients included in the final analysis

Institution	Excisional surgery	Pit-picking and variations	Total	Included in the study
Istanbul Medipol University	65	372	437	156
Acibadem University	270	161	431	69
Medicana Bursa Hospital	213	1807	2020	33
Medical Park Gebze Hospital	660	180	840	33
Cukurova University	334	90	424	13
Dokuz Eylul University Hospital	210	152	362	9
Memorial Sisli Hospital	25	55	80	2
Overall	1777	2817	4594	306

### Clinical characteristics

The median age was 24 (range 18–54) years; 193 (63.1%) patients were male and 113 (36.9%) female. Male patients were significantly more likely to receive combined treatment (68.9%) than females (56.6%, *p* = 0.021). The groups were similar regarding age, BMI, symptom duration, and abscess history (Table 2). Forty-four (14.4%) patients had Tezel V (recurrent) PSD. Recurrent presentation was more common in the pit-picking + LT group (18.3%) than in the pit-picking group (7.3%) (*p* < 0.001) (Table 2).

**Table 2:** Demographic and clinical characteristics of patients

	Total (n = 306)	Pit-picking (n = 109, 35.6%)	Pit-picking + LT (n = 197, 64.4%)	p
Age (years, median, range)	24 (18–54)	25 (18–49)	24 (18–54)	0.507
Sex				0.021
Female	113 (36.9)	49 (43.4)	64 (56.6)	
Male	193 (63.1)	60 (31.1)	133 (68.9)	
Family history (+)	41 (13.4)	18 (16.5)	23 (11.7%)	0.155
BMI (kg/m^2^, mean ± SD)	26 ± 3.4	26.1 ± 3.2	25.9 ± 3.4	0.629
Duration of the symptoms (months, median, range)	8 (1–108)	10 (1–108)	7 (1–72)	0.407
History of abscess drainage	79 (25.8)	33 (30.3)	46 (23.4)	0.118
Smoking (+)	135 (44.4)	47 (43.1)	88 (45.1)	0.414
Tezel Classification				< 0.001
III	183 (59.8)	59 (54.1)	124 (62.9)	
IV	79 (25.8)	42 (38.5)	37 (18.8)	
V	44 (14.4)	8 (7.3)	36 (18.3)	

LT: laser treatment, SD: standard deviation, BMI: body mass index

Most procedures were performed under local anesthesia (84.6%), with a significantly higher proportion in the pit-picking group (96.3% vs. 78.2%, < 0.001). The mean operative time was similar between groups (24.1 ± 5.7 vs. 22.9 ± 8.3 min, *p* = 0.172) (Table 3).

**Table 3:** Comparison of surgical characteristics and outcomes

	Total (n = 306)	Pit-picking (n = 109, 35.6%)	Pit-picking + LT (n = 197, 64.4%)	p
Anesthesia				< 0.001
Local	259 (84.6)	105 (96.3)	154 (78.2)	
General	36 (11.8)	4 (3.7)	32 (16.2)	
Spinal	0	11 (5.6)	11 (3.6)	
Operative time (min, mean ± SD)	23.4 ± 7.4	24.1 ± 5.7	22.9 ± 8.3	0.172
Complications	28 (9.2)	16 (14.7)	12 (6.1)	0.012
Seroma	16 (5.2)	12 (11)	4 (2)	0.001
Bleeding	8 (2.6)	7 (6.4)	1 (0.5)	0.004
Surgical site infection	9 (2.9)	3 (2.8)	6 (3)	0.594
Time to return to work (days, median, range)	5 (0–20)	6 (3–17)	3 (0–20)	< 0.001
Time to sit pain-free (days, median, range)	6 (1–17)	7 (3–17)	5 (1–17)	< 0.001
Time to complete healing (days, median, range)	14 (2–60)	14 (8–30)	14 (2–60)	0.083
Pain score (VAS, median, range)				
24 h	2.5 (0–10)	3 (1–7)	2 (0–10)	0.265
7 days*	1 (0–3)	1 (0–3)	0 (0–3)	< 0.001
30 days*	0 (0–1)	0 (0–1)	0 (0–1)	0.832
Follow-up (months, median, range)	68 (60–92)	65 (60–86)	70 (60–92)	< 0.001
Recurrence (*n*,%)	40 (13.1)	15 (13.8)	25 (12.7)	0.460
Time-to-recurrence (days, median, range)	15 (1–90)	15 (2–24)	14.5 (1–90)	0.990

LT: laser treatment SD: standard deviation, VAS: visual analog scale, *analyzed for 156 patients whose data were available

### Complications

Overall complications were seen in 28 (9.2%) patients, including 16 seromas, 8 bleedings, and 9 surgical site infections, which were all managed conservatively. The complication rate was higher in the pit-picking group (14.7%) compared to the pit-picking + LT group (6.1%, *p* = 0.012). Seroma formation (11% vs. 2%, *p* = 0.001) and bleeding were significantly more common in the pit-picking group (6.4% vs. 0.5%, *p* = 0.004), where surgical site infection rates were similar between groups (2.8% vs 3%, *p* = 0.594) (Table 3). 

### Return to work, pain, and complete healing

The time to return to work (median 3 days vs. 6 days, *p* < 0.001) and time to sit pain-free (5 days vs. 7 days, *p* < 0.001) were significantly shorter in the pit-picking + LT group. The VAS score at 24 h was a median of 2.5 (0–10) and similar between groups. On day 7, the median VAS score was 0 (0–3) for the pit-picking + LT group and significantly lower than the pit-picking group (median 1, [0–3], *p* < 0.001) (Table 3).

Complete healing was achieved at 97.4% of the entire series. Time to complete healing was a median of 14 (2–60) days and similar between groups (*p* = 0.083). Eight (2.6%) patients (4 in each groups) had persistent symptoms for 2 months, and they were managed as recurrent patients (Table 3).

### Recurrence

**Fig. 3:**
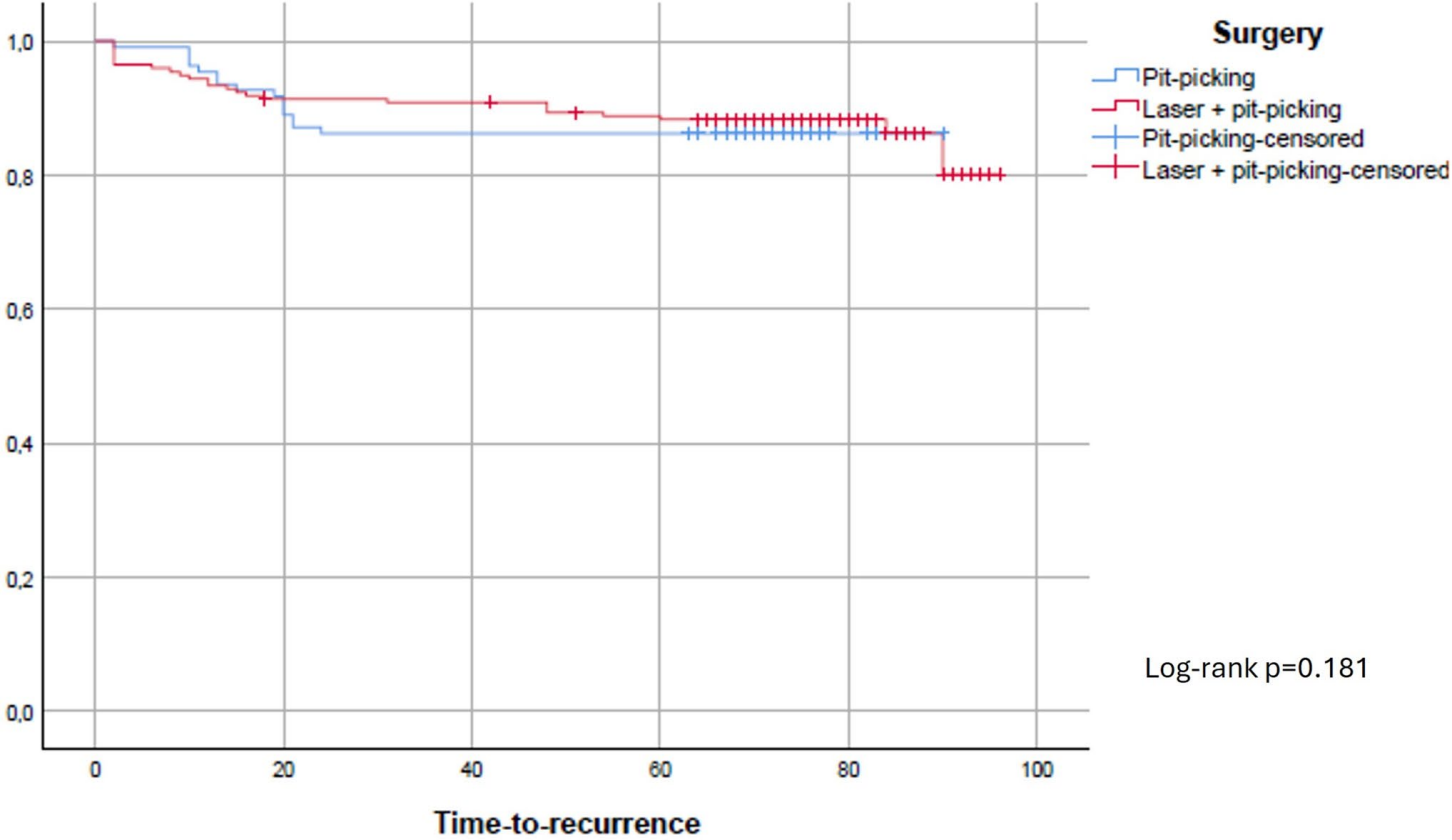
Kaplan-Meier curves for time to recurrence comparing the pit‑picking (blue) versus pit‑picking with laser (red) groups, with tick marks indicating censored observations; no significant difference (log‑rank *p* = 0.181)

**Fig. 4:**
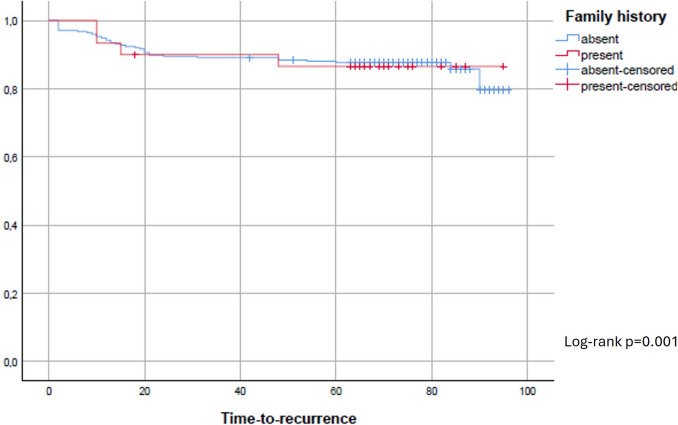
Kaplan-Meier curves for time to recurrence by family history status, comparing absent (blue) versus present (red), with tick marks indicating censored observations; a significant difference was observed (log‑rank *p* = 0.001)

**Fig. 5:**
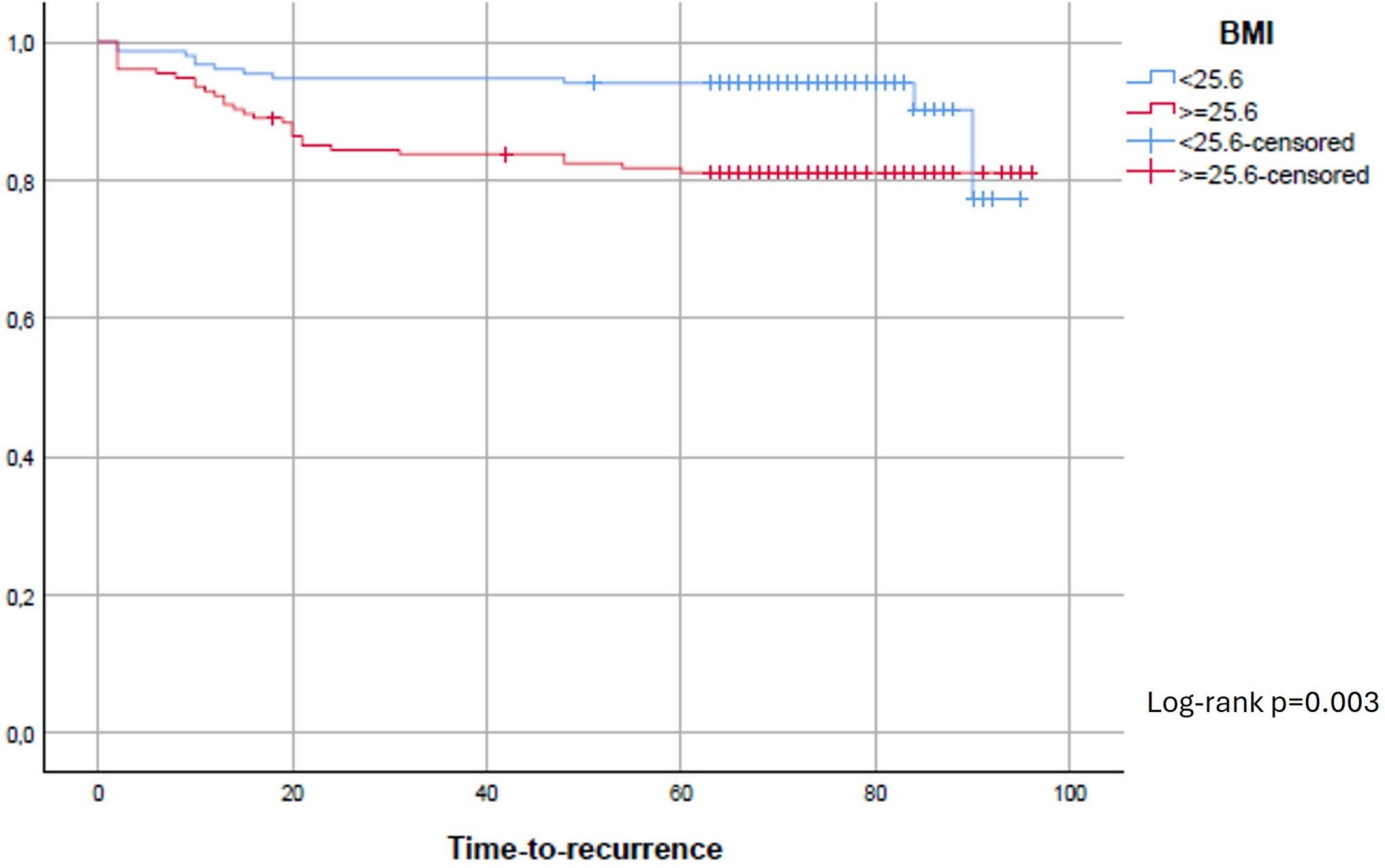
Kaplan-Meier curves for time to recurrence by BMI category, comparing < 25.6 (blue) versus ≥ 25.6 (red), with tick marks indicating censored observations; a significant difference was observed (log‑rank *p* = 0.003)

**Fig. 6:**
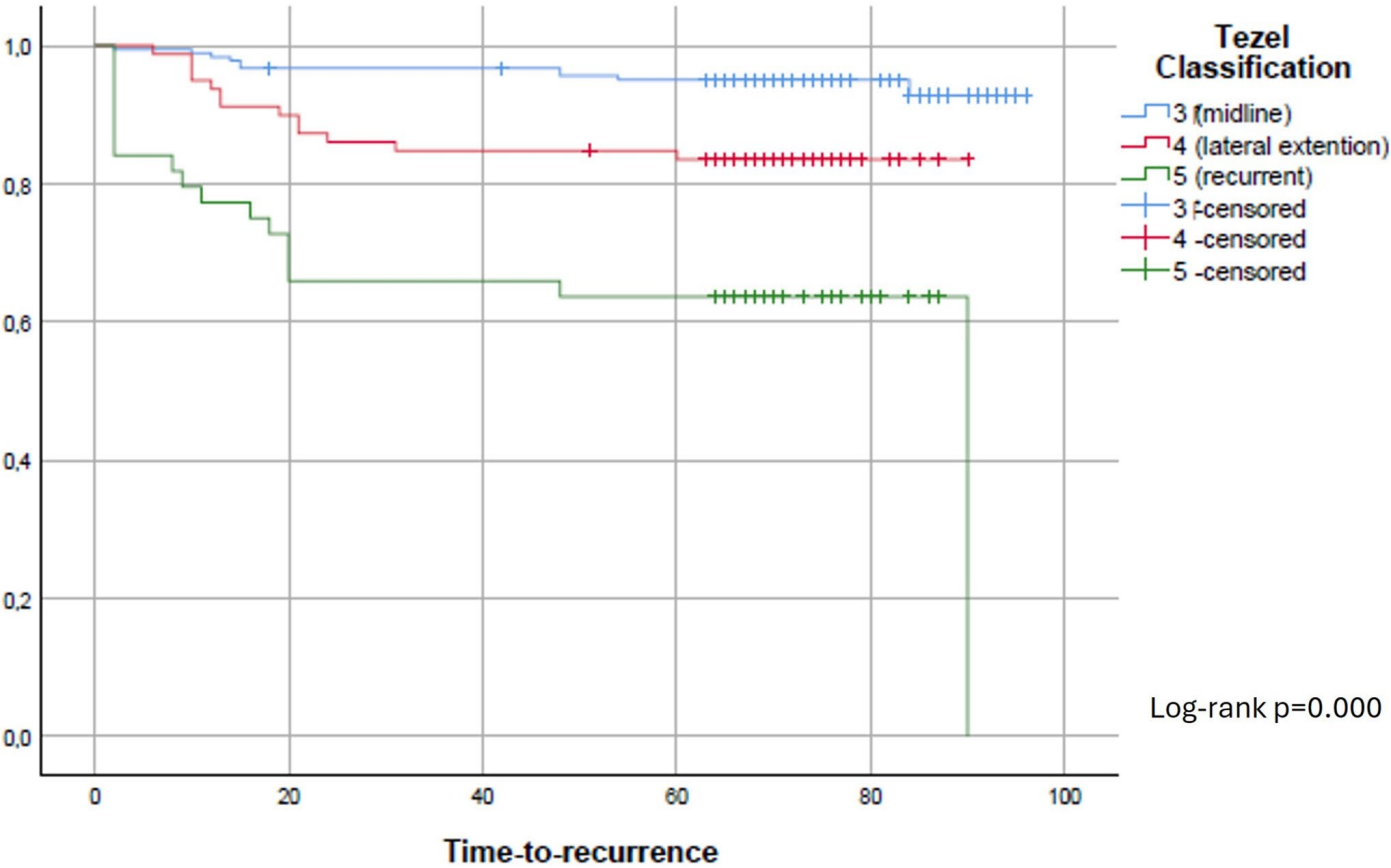
Kaplan-Meier curves for time to recurrence by Tezel classification, comparing class 3 (midline, blue), class 4 (lateral extension, red), and class 5 (recurrent, green), with tick marks indicating censored observations; a significant difference was observed (log‑rank *p* < 0.001)

**Fig. 7:**
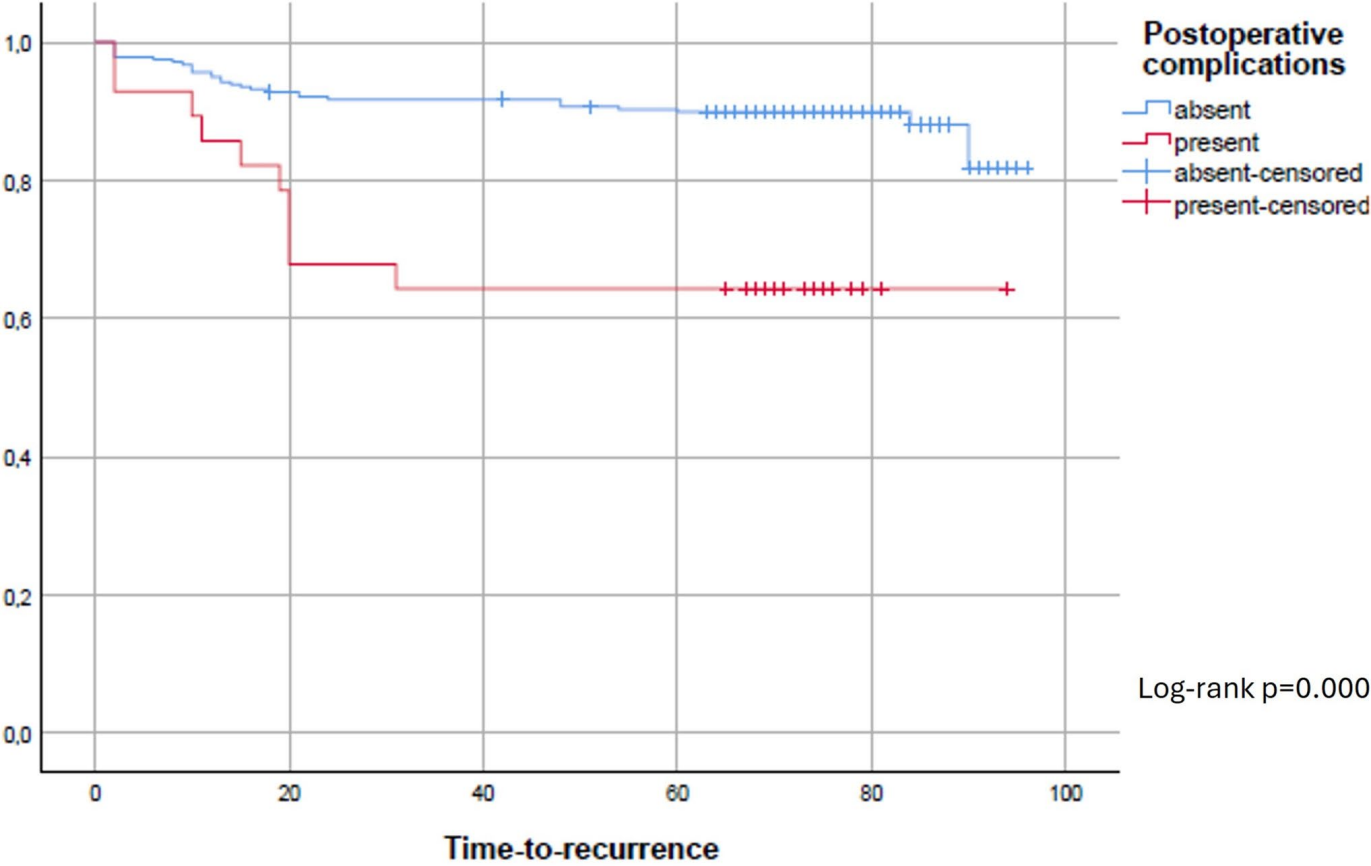
Kaplan-Meier curves for time to recurrence by postoperative complication status, comparing absent (blue) versus present (red), with tick marks indicating censored observations; a significant difference was observed (log‑rank *p* < 0.001)

The median follow-up was 68 (60–92) months. Recurrence was seen in 40 (13.3%) patients: 3 (23%) at Cukurova University, 6 (18.2%) at Medical Park Gebze Hospital, 10 (14.5%) at Acibadem University, 20 (12.8%) at Medipol University, and 1 (3%) at Medicana Bursa Hospital. The median time to recurrence was 15 (1–90) months. Recurrence rates at 5 years were similar between the two groups (pit-picking 13.8% vs. pit-picking + LT 12.7%, *p* = 0.460) (Table 3, Fig. 3). Patients with a family history of PSD had a recurrence rate of 32.5%, which was significantly higher compared with those without a family history (10.5%, *p* = 0.001) (Fig. 4). The mean BMI (28.2 in recurrent vs. 25.5 in non-recurrent patients, *p* < 0.001) (Fig. 5), Tezel classification (*p* < 0.001) (Fig. 6), and complications (Fig. 7) were other factors associated with recurrence. Recurrence rates for Tezel III, IV, and V patients were 5.5%, 16.5%, and 38.6%, respectively. Patients who had postoperative complications (25%) were more likely to have recurrence than those who did not (6.8%) (*p* = 0.001) (Table 4).

**Table 4:** Comparison of demographic and clinical characteristics between patients with and without recurrence at 5 years

	Recurrence (−) (n = 266)	Recurrence (+) (*n* = 40)	p
Age (years, median, range)	24 (18–54)	25 (18–47)	0.559
Sex			0.395
Female	97 (85.8)	16 (14.2)	
Male	169 (87.6)	24 (12.4)	
BMI (kg/m^2^, median, range)	25.5 (17.5–37.1)	28.2 (23–37.1)	< 0.001
Family history (+)	13 (32.5)	28 (10.5)	0.001
Duration of the symptoms (months, median, range)	8 (1–72)	10 (2–108)	0.582
History of abscess drainage (+)	67 (84.8)	12 (15.2)	0.318
Smoking (+)	121 (89.6)	14 (10.4)	0.132
Tezel Classification			< 0.001
III	172 (94.5)	10 (5.5)	
IV	66 (83.5)	13 (16.5)	
V	27 (61.4)	17 (38.6)	
Overall complications (+)	18 (6.8)	10 (25)	0.001

### Laser devices and energy

Laser treatment was performed using the Biolitec device in 82 patients (41.6%) and the NeoV device in 115 patients (58.4%). The wavelength was 980 nm in 13 patients (4.2%) and 1470 nm in 184 patients (93.4%). The power setting was 15 W in 13 patients (6.6%), 13 W in 69 patients (35%), and 10 W in 115 patients (58.4%). Recurrence rates did not differ according to the device, power, or wavelength used (Table 5).

**Table 5:** Recurrence rates according to device, energy, and wavelength in laser-treated patients

	Recurrence (−) (n = 172)	Recurrence (+) (*n* = 25)	p
Laser device			0.315
Biolitec	70 (85.4%)	12 (14.6%)	
NeoV	102 (88.7%)	13 (11.3%)	
Energy			0.784
10 W	102 (88.7%)	13 (11.3%)	
13 W	59 (85.5%)	10 (14.5%)	
15 W	11 (84.6%)	2 (15.4%)	
Wave length			0.511
980 nm	11 (84.6%)	2 (15.4%)	
1470 nm	161 (87.5%)	23 (12.5%)	

### Cost analysis

The total hospital costs were significantly higher in the pit-picking + LT group compared with the pit-picking group ($1212 ± 146 vs. $888 ± 148, p = 0.004). Among these 133 patients, 10 experienced postoperative complications, including 7 cases of seroma and 3 cases of abscess, all of which occurred in the pit-picking group. Seromas were managed through repeated aspirations until complete healing, while abscesses were drained in the office setting. These patients required an average of 5.5 ± 3.2 outpatient office visits. When all costs—those related to office visits, wound care, and seroma aspirations—were included, the mean cost for patients with complications increased to $1198 ± 370 (Table 6).

**Table 6:** Total hospital costs including procedure-related and 60-day follow-up-related charges. The cost-analysis was conducted in a subgroup of 133 patients operated on under local anesthesia and discharged on the same day at the same center representing similar insurance and reimbursement policies

*n* = 133*	Pit-picking (n = 80)	Pit-picking + LT (*n* = 53)	p
Total costs (USD, mean ± SD)	888 ± 148	1212 ± 146	0.004
Patients with complication	1198 ± 370, n = 10	n = 0	
Patients without complication	818 ± 115, n = 70	1212 ± 146	< 0.001

SD: standard deviation, LT: laser treatment, *patients from Istanbul Medipol University

### Post hoc power analysis

To quantify the probability of detecting the observed between-group difference in 5-year recurrence, we conducted a post hoc power analysis for two independent proportions (two-sided α = 0.05). Using the observed incidences (pit-picking: 13.8%, *n* = 109; pit-picking + laser: 12.7%, *n* = 197), the absolute difference was 1.1 percentage points. Power was computed with a z-test for proportions under the observed effect, employing the pooled variance under the null for the test threshold and the group-specific variances under the alternative; equivalently, the calculation can be expressed via Cohen’s h (h = 2·arcsin√p1 − 2·arcsin√p2). The achieved power was 4.7%, indicating a very low probability of detecting such a small difference with the available sample sizes. Accordingly, the non-significant result should be interpreted as limited power to detect small effects rather than definitive evidence of no difference.

## Discussion

Our findings indicate that the addition of LT to pit-picking does not significantly impact long-term recurrence rates. However, the LT group had fewer postoperative complications, less pain on day 7, achieved pain-free sitting earlier, and returned to work earlier. Factors contributing to recurrence were high BMI, family history, advanced Tezel stage, and postoperative complications. Despite the evident early clinical benefits, the costs were significantly higher in the LT group. Moreover, we observed that whether combined with LT or not, pit-picking has comparable long-term recurrence rates to primary open methods [16].

Excisional surgery remains the standard treatment, with reported 5-year recurrence rates of approximately 10% for off-midline techniques [1, 2, 17], which was comparable to our results. The 5-year recurrence rate of 13.1% with pit-picking was not affected by the addition of LT in our series. Factors associated with recurrence after PSD surgery are not well established. While some studies have shown that BMI is associated with higher recurrence rates [18, 19], others have suggested the opposite [16]. Young age, family history, hair structure, bathing habits, smoking, duration of the symptoms, history of abscess drainage, prolonged sitting, and a deep natal cleft are other proposed factors; however, the results remain contradictory and do not report high-quality evidence [19–23]. Since our data were limited regarding factors potentially influencing recurrence, including hair structure, perioperative hair removal, and perioperative antibiotic prophylaxis, we did not perform a multivariable analysis. However, based on the available data, recurrence was more frequent in patients with high BMI, family history, and more severe disease in our series.

Minimally invasive treatments are recommended for mild cases in guidelines and consensus reports [24–27]. In line with this, our study found recurrence rates of 5.5% for Tezel III, 16.5% for Tezel IV, and 38.6% for Tezel V patients. Although our results do not demonstrate highly favorable outcomes for LT in recurrent (Tezel V) disease, a recent meta-analysis conducted by Qin et al. [10] reported a healing rate of 81.9%, which decreased to 74.5% when follow-up duration exceeded 12 months. Our median follow-up was 70 months, and the healing rate was 61.4% in recurrent presentation. As reported in the comprehensive meta-analysis by Stauffer et al. [1], an increase in recurrence rates with longer follow-up is expected. These results along with other literature suggest that minimally invasive methods can be a viable option in recurrent PSD [6, 10].

Literature on LT remains limited, but a recent review including 971 patients who underwent LT reported a recurrence rate of 3.8% with a median follow-up of 12 months [5]. The effect of LT when added to pit-picking has not been evaluated before; however, Qin et al. [10] reported the outcome of two studies reporting LT without pit-picking and concluded that the combination of pit-picking with LT did not affect recurrence rates. The complication rates after LT have been reported in two relatively large studies. De Decker [28] reported 8% wound infection and a mean of 41 days to complete healing, whereas Georgiou [29] reported a 10.4% complication rate with a mean of 4.5 days for return to activity. In our series, patients in the LT group experienced more than a twofold reduction in complication rates (6.1% vs. 14.7%) and a shorter time to return to work (3 days vs. 6 days). Moreover, a greater proportion of patients in the LT group were male and classified as Tezel V, both of which may increase susceptibility to complications. LT may reduce early complications through thermal coagulation and gentle tissue contraction, sealing the microvasculature and shrinking residual cavities.

In our cost analysis, we found that the cost for patients with complications in the pit-picking group was similar to that of patients in the LT group. Unfortunately, our cost analysis does not account for workforce loss or the societal burden. When considering the cost of LT, the potential cost benefits of early return to work and the postoperative comfort it provides should be taken into account. Future studies should focus on evaluating the financial burden of LT and cost-effectiveness in greater detail.

### Limitations

The most important limitation of our study was its retrospective design and small sample size. This modest sample and event rate may limit the power to detect small differences in 5-year recurrence. The post hoc power for the observed 1.1-percentage-point difference was only 4.7% at α = 0.05. Consequently, the lack of a statistically significant between-group difference should be interpreted with caution, as the study was underpowered to detect subtle effects. In interpreting early outcomes, the LT group had more Tezel V and the pit-picking group more Tezel IV; this imbalance may influence pain and complications; however Tezel III (simple) disease was similarly distributed. Additionally, the number of treated sinuses was not consistently recorded across all patients, which may further confound these comparisons. A key limitation is the absence of multivariable adjustment (e.g., Cox or logistic regression) to account for confounders such as BMI and Tezel stage, which we avoided because of incomplete covariate data (hair removal, antibiotic use). Our cohort included a higher proportion of female patients (36.9%) than in national reports (25%) [30], which may introduce bias. This may have been influenced by the fact that the two operating surgeons were female. The heterogeneity in the number of patients included across centers also limits the generalizability of our results. The cost analysis was limited to a single center and a specific payment model (a private hospital with a public co-payment). The conclusions on cost may not be transferable to other healthcare systems.

## Conclusion

Pit-picking, with or without LT, is a safe and effective treatment for PSD. The addition of LT does not affect long-term recurrence and cost increases. However, the improved early postoperative outcomes may justify the use of LT. A thorough cost-benefit analysis should be conducted and shared with patients when considering LT. Further randomized trials are necessary to refine patient selection and treatment indications.

## Data Availability

No datasets were generated or analysed during the current study.

## Declarations

### Clinical Trial Registration

NCT05569135, Registration date: 05.10.2022.
